# Metaplastic breast carcinomas exhibit EGFR, but not HER2, gene amplification and overexpression: immunohistochemical and chromogenic *in situ *hybridization analysis

**DOI:** 10.1186/bcr1341

**Published:** 2005-10-25

**Authors:** Jorge S Reis-Filho, Fernanda Milanezi, Silvia Carvalho, Pete T Simpson, Dawn Steele, Kay Savage, Maryou BK Lambros, Emilio M Pereira, Jahn M Nesland, Sunil R Lakhani, Fernando C Schmitt

**Affiliations:** 1 The Breakthrough Breast Cancer Research Centre, Institute of Cancer Research, London, UK; 2 IPATIMUP – Institute of Molecular Pathology and Immunology, University of Porto, Porto, Portugal; 3 Life and Health Sciences Research Institute (ICVS), School of Health Sciences, University of Minho, Braga, Portugal; 4 Molecular & Cellular Pathology, Mayne Medical School, University of Queensland, Queensland Institute of Medical Research and Royal Brisbane and Women's Hospital, Brisbane, Australia; 5 Laboratório Salomão & Zoppi, São Paulo, Brazil; 6 The Norwegian Radium Hospital, University of Oslo, Montebello, Norway

## Abstract

**Introduction:**

Metaplastic breast carcinomas constitute a heterogeneous group of neoplasms, accounting for less than 1% of all invasive mammary carcinomas. Approximately 70–80% of metaplastic breast carcinomas overexpress the epidermal growth factor receptor (EGFR). Human epidermal growth factor receptor (HER)2 and EGFR have attracted much attention in the medical literature over the past few years owing to the fact that humanized monoclonal antibodies against HER2 and therapies directed against the extracellular ligand-binding domain or the intracellular tyrosine kinase domain of EGFR have proven successful in treating certain types of human cancer. We investigated whether HER2 and EGFR overexpression was present and evaluated gene amplification in a series of metaplastic breast carcinomas.

**Method:**

Twenty-five metaplastic breast carcinomas were immunohistochemically analyzed using a monoclonal antibody (31G7) for EGFR and two antibodies for HER2 (Herceptest and CB11) and scored using the Herceptest scoring system. Gene amplification was evaluated by chromogenic *in situ *hybridization using Zymed Spot-Light EGFR and HER2 amplification probe. The results were evaluated by bright field microscopy under 40× and 63× objective lenses.

**Results:**

Nineteen (76%) metaplastic breast carcinomas exhibited EGFR ovexpression, and among these EGFR amplification (defined either by large gene clusters or >5 signals/nucleus in >50% of neoplastic cells) was detected in seven cases (37%): three carcinomas with squamous differentiation and four spindle cell carcinomas. One case exhibited HER2 overexpression of grade 2+ (>10% of cells with weak to moderate complete membrane staining), but HER2 gene amplification was not detected.

**Conclusion:**

Metaplastic breast carcinomas frequently overexpressed EGFR, which was associated with EGFR gene amplification in one-third of cases. Our findings suggest that some patients with metaplastic breast carcinomas might benefit from novel therapies targeting EGFR. Because most metaplastic breast carcinomas overexpress EGFR without gene amplification, further studies to evaluate EGFR activating mutations are warranted.

## Introduction

In recent years, the family of epidermal growth factor receptors (EGFRs) or ERBB receptors has attracted great attention in the literature [1]. This family includes four tyrosine kinase receptors: EGFR (HER1/c-erbB1), human epidermal growth factor receptor (HER)2/neu (c-erbB2), HER3 (c-erbB3) and HER4(c-erbB4). All members of this family are characterized by an extracellular ligand-binding region, a single membrane spanning region, and a cytoplasmic tyrosine kinase containing domain [1]. Although expression of the four members of the ERBB family has been studied in several types of human tumours [1], only EGFR and HER2 have been proven to play major roles in different histological types of breast cancer [1-12]. Thus far, only these two receptors have successfully been targeted as therapy for lung [13-17] and breast cancer [18,19].

EGFR was the first tyrosine kinase receptor to be directly linked with human cancer. The EGFR gene maps to 7p11.2-p2 and encodes a 170 kDa transmembrane protein [1]. EGFR gene amplification has been described in oligodendrogliomas [20], glioblastomas [21], lung carcinomas [13,14,22], gastric carcinomas [23] and, recently, breast carcinomas [8,24,25]. The *HER2* gene maps to chromosome 17q21 and encodes a 185 kDa glycoprotein. It is reported to be amplified and overexpressed in several types of human tumours, including about 30% of all breast carcinomas [1,18,19]. Most importantly, in recent years EGFR tyrosine kinase inhibitors and humanized monoclonal antibodies against HER2 have received US Food and Drug Administration approval and are currently being tested in patients with lung and breast cancer.

Data on the response of patients with lung cancer have demonstrated that approximately 10–15% of patients with EGFR-positive lung carcinomas have a dramatic response to EGFR tyrosine kinase inhibitors [15-17,22]. Interestingly, response was linked to the presence of an activating somatic mutation targeting the tyrosine domain of EGFR [15-17]. In addition, Cappuzzo and coworkers [22] demonstrated that EGFR amplification is a strong predictor of response to EGFR tyrosine kinase inhibitors. Unlike EGFR activating mutations, EGFR amplifications also exhibited a statistically significant association with survival [22].

Although *HER2* gene amplification and protein overexpression have been extensively studied in breast cancer, data on *EGFR* amplification in breast cancer are limited. HER2 overexpression has been identified in 87% and 27% breast carcinomas with EGFR overexpression [7] and gene amplification [8], respectively. Interestingly, EGFR and HER2 coexpression in breast cancer was recently associated with reduced overall survival (OS) and disease-free survival (DFS) [7].

Our group and others have demonstrated that 'basal-like' breast carcinomas and metaplastic breast carcinomas (MBCs) consistently overexpress EGFR but usually lack HER2 overexpression [12,26]. However, the presence of *EGFR* gene amplifications have not been systematically analyzed in a series of MBCs. The aims of the present study were threefold: to analyze the presence of *EGFR* gene amplifications in MBCs; to correlate the presence of *EGFR* amplifications with EGFR immunohistochemical overexpression; and to assess HER2 overexpression in MBCs.

## Materials and methods

### Metaplastic breast carcinoma samples

Cases of MBC were identified and samples retrieved from the pathology files of the Royal Marsden Hospital (London, UK), the Institute of Molecular Pathology and Immunology, University of Porto (Porto, Portugal), and the Norwegian Radium Hospital (Montebello, Norway). The project was approved by the local ethics committees.

Cases of MBC were identified from the Royal Marsden from January 1980 to March 2004 by searching the electronic Hospital Information System for cases diagnosed as adenosquamous carcinoma, carcinosarcoma, metaplastic breast carcinoma, sarcomatoid carcinoma, spindle cell carcinoma and squamous cell carcinoma, as well as from the consultation files of one of the authors (SRL). Cases from the Institute of Molecular Pathology and Immunology and the Norwegian Radium Hospital were identified from the consultation files of two of the authors (JMN and FCS, respectively). All cases were initially reviewed by the contributing authors, who evaluated additional immunohistochemical markers to corroborate the diagnosis.

The cases were centrally reviewed by three of the authors (JSR-F, FM and FCS) on a multiheaded microscope and classified according to previously described criteria [27-33]. Briefly, tumours were classified as matrix producing breast carcinomas if chondroid and/or osseous matrix was observed in the absence of spindle and osteoclast giant cell components [31]. Neoplasms were classified as spindle cell carcinomas if intraductal or infiltrating ductal or squamous carcinoma of ductal origin was contiguous or subtly merged with a spindle cell proliferation of neoplastic cells, which comprised at least 50% of the tumour bulk [30]. Carcinomas with heterologous elements were defined as tumours with an intraductal or invasive carcinomatous component intimately admixed or subtly merging with a sarcomatous spindle cell component with evidence of chondroid, osseous, or rhabdomyoid differentiation [29,32]. Carcinomas with squamous differentiation were predominantly (>50%) or completely composed of apparent squamous cell components admixed with areas of invasive ductal and/or spindle cell carcinoma, in the absence of involvement of the ovevrlying skin [28,33]. A median of two representative blocks from each case was selected for immunohistochemical and chromogenic *in situ *hybridization (CISH) analysis.

### Immunohistochemical and chromogenic *in situ *hybridization analysis

Immunohistochemistry was performed with antibodies raised against HER2 (Herceptest^®^(Dako, Glosatrup, Denmark), polyclonal, 1/10 epitope retrieval solution (Dako) at 98°C, prediluted; and CB11 (Novocastra, Newcastle-upon-Tyne, UK), 2 min in a pressure cooker, 1:100) and EGFR (31G7, 1:50, Zymed (South San Francisco, CA, USA)), as previously described [34]. For Her2/neu and EGFR, the Herceptest^® ^scoring system was applied: negative = no membrane staining or <10% of cells stained; 1+ = incomplete membrane staining in >10% of cells; 2+ = >10% of cells with weak to moderate complete membrane staining; and 3+ = strong and complete membrane staining in >10% of cells.

CISH was performed using Spot-Light amplification probes for *EGFR* (Zymed) and *HER2* (Zymed), in accordance with the manufacturer protocol and as previously described [34]. Because the interpretation guidelines for Spot-Light *EGFR* and *HER2* amplification probes have previously been validated [8,34,35], we did not use α-satellite probes for chromosomes 7 and 17, respectively. All cases were subjected to CISH for *EGFR*, and only those with HER2 grade 2+ or 3+ positivity were subjected to CISH for *HER2*[36]. Appropriate gene amplified breast tumour controls were included in each run. Each section was analyzed by two of the authors (FM and SC) on a multiheaded microscope. Only unequivocal signals were counted. Signals were evaluated at 400× and 630×, and at least 60 cells were evaluated for the presence of the *EGFR* probe. A given tumour was considered to be amplified for *EGFR* or *HER2* when more than 50% of the neoplastic cells exhibited more than five signals per nuclei or large gene signal clusters [8,34,36].

Out of the 112 cases of metaplastic breast carcinomas in our series, 25 had sufficient material in the blocks and were successfully analyzed by both immunohistochemistry and CISH for EGFR and HER2.

### Correlation between EGFR overexpression and amplification and clinicopathological parameters and survival

Follow-up information was available for 23 out of 25 patients, with follow-up periods ranging from 5.5 to 124.3 months (median 34.6 months, mean 51.9 months). The Statview software package was used for all calculations. Correlations between categorical variables were performed using the χ^2 ^test and Fisher's exact test. Correlations between continuous and categorical variables were performed with analysis of variance. DFS and OS were expressed as the number of months from diagnosis to the occurrence of an event (local recurrence/metastasis and disease-related death, respectively). Cumulative survival probabilities were calculated using the Kaplan–Meier method. Differences between survival rates were tested using the log-rank test. All tests were two-tailed, with a confidence interval of 95%.

## Results

**Figure 1:**
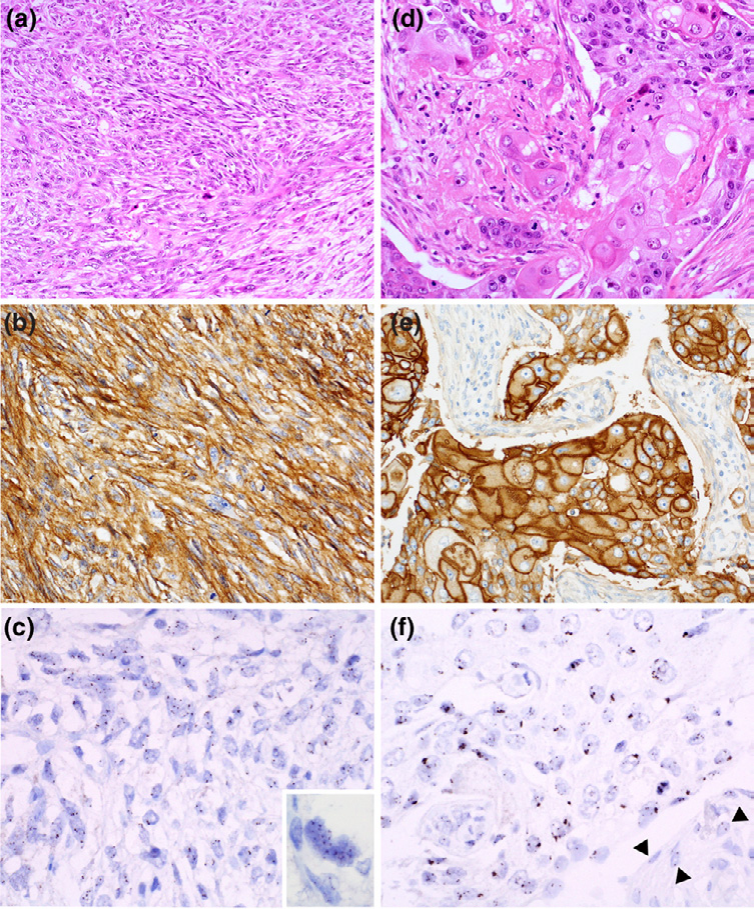
EGFR overexpression and gene amplification in MBCs. Photomicrographs of **(a) **a spindle cell metaplastic breast carcinoma (haematoxylin and eosin) showing **(b) **grade 3+ immunohistochemical positivity for EGFR and **(c) ***EGFR* gene amplification (>5 signals per nucleus [CISH]). Inset in panel c: note the bizarre neoplastic cell with more than 10 copies of *EGFR*. **(d) **Breast carcinoma with squamous metaplasia (haematoxylin and eosin) with **(e) **EGFR grade 3+ immunohistochemical positivity. **(f) **CISH demonstrating *EGFR* amplification (clusters of signals in the nuclei of neoplastic cells). Note the presence of one or two signals in the nuclei of stromal cells (arrowheads). CISH, chromogenic *in situ *hybridization; EGFR, epidermal growth factor receptor; MBC, metaplastic breast carcinoma.

**Figure 2:**
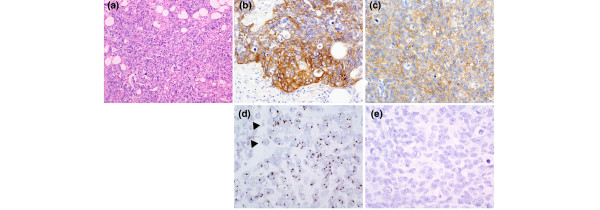
EGFR and HER2 overexpression and gene amplification in a spindle cell carcinoma. **(a) **Photomicrograph of a spindle cell carcinoma (haematoxylin and eosin). Immunohistochemical analysis revealed **(b) **EGFR grade 3+ positivity and **(c) **HER2 grade 2+ reactivity. **(d) **CISH demonstrating *EGFR* amplification (clusters of signals in the nuclei of neoplastic cells). Note the presence of one or two copies of *EGFR* in stromal cells (arrowheads). **(e) **CISH for *HER2* gene: no amplification (2–3 gene copies/nucleus). CISH, chromogenic *in situ *hybridization; EGFR, epidermal growth factor receptor; HER, human epidermal growth factor receptor; MBC, metaplastic breast carcinoma.

Table 1 summarizes the results of the histological, immunohistochemical and CISH analyses. Briefly, 19 out of 25 (76%) cases exhibited EGFR positivity of grade 3+. No samples showed EGFR grade 2+ positivity, whereas four cases were EGFR 1+. Out of the 19 cases with EGFR grade 3+ expression, seven (37%) exhibited *EGFR* gene amplification (Fig. 1). Three out of nine carcinomas with squamous metaplasia and four out of 10 spindle cell carcinomas had *EGFR* amplification, whereas no matrix producing breast carcinomas showed any amplification. Interestingly, similar numbers of *EGFR* signals were observed in the epithelial and in the metaplastic elements. One case exhibited HER2 grade 2+ positivity with Herceptest^®^, but *HER2* gene amplification was not observed (Fig. 2).

**Figure 3:**
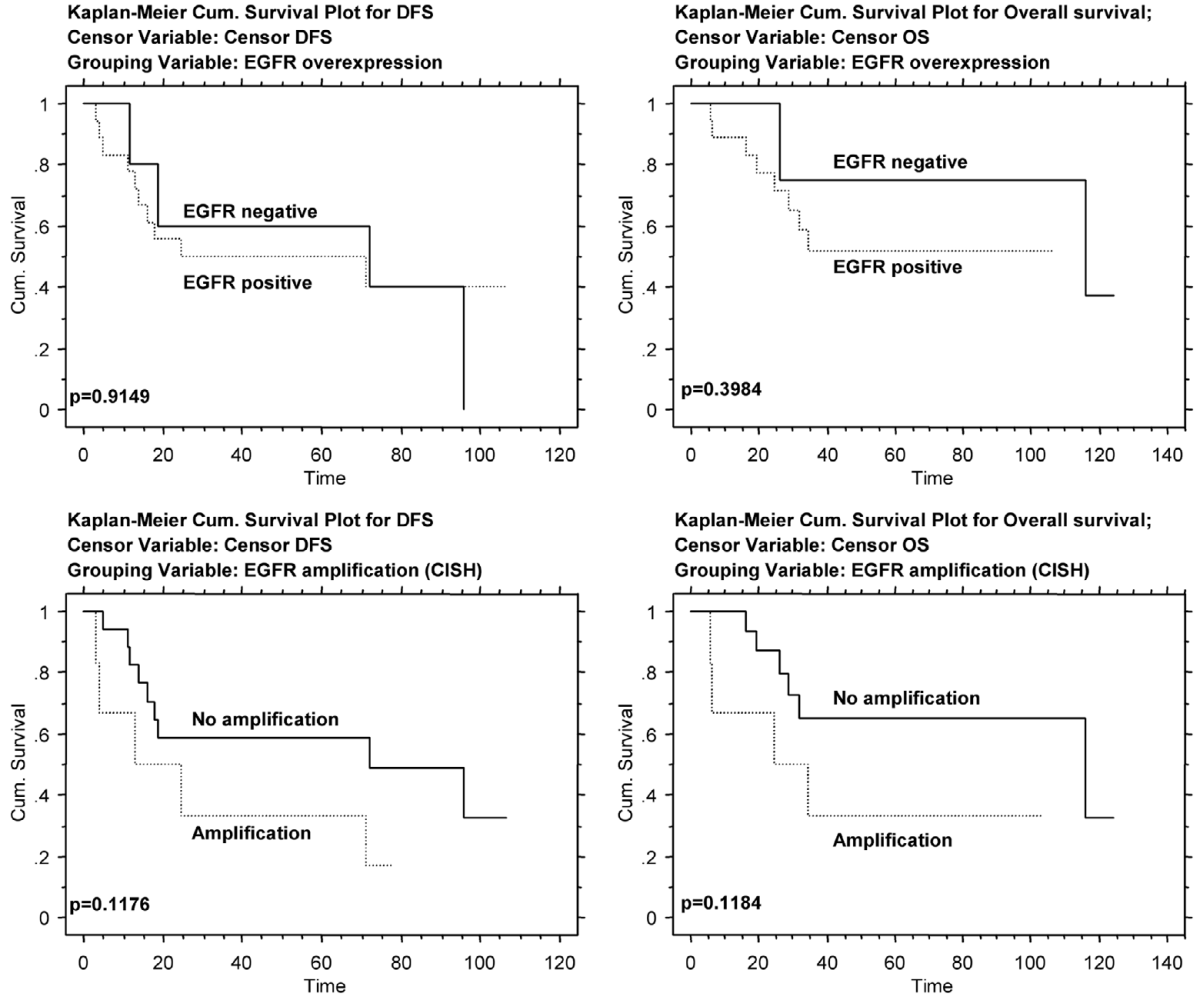
EGFR overexpression and amplification: prognostic impact on DFS and OS. CISH, chromogenic *in situ *hybridization; DFS, disease-free survival; EGFR, epidermal growth factor receptor; OS, overall survival.

No association between EGFR overexpression or amplification and clinicopathological features was observed (Table 2). EGFR overexpression showed no association with DFS or OS. Patients with tumours harbouring EGFR amplification had a trend toward shorter DFS and OS (Fig. 3).

## Discussion

In recent studies, we and others demonstrated that MBCs frequently overexpress EGFR and lack HER2 overexpression. In a previous study [26] we demonstrated that up to 83% of all MBCs show EGFR overexpression. Leibl and Moinfar [12] described positivity for EGFR in 70% of MBCs, but those authors also considered cases with grade 1+ expression to be positive. When only grade 2+ and 3+ expression was considered to represent positivity, 60% (12/20) were positive. In the present study we demonstrated that 76% (19/25) of MBCs overexpressed EGFR. The differences between our findings and those of Leibl and Moinfar [12] may be related to the different antibody clones used and different antigen retrieval methods.

The mechanism underlying EGFR overexpression has not been investigated in MBCs. In the present study we demonstrated that *EGFR* is amplified in 28% (7/25) of MBCs and in 37% (7/19) of MBCs with EGFR overexpression. Although only six cases with heterologous elements (four matrix producing carcinomas and two carcinomas with heterologous elements) were analyxed, no amplification was found in these two subtypes of MBC. Identification of areas of squamous differentiation in spindle cell carcinomas and the presence of spindle cells in carcinomas with squamous metaplasia are not infrequent. In fact, in the seminal study conducted by Huvos and coworkers [32] these two subtypes of MBCs were classified under the heading 'group 1' MBCs. In addition, when EGFR is overexpressed in carcinomas with heterologous elements and matrix producing breast carcinomas, its expression appears to be more conspicuous in epithelial components (data not shown). In a recent study, Bhargava and coworkers [8] demonstrated that 6% (11/175) of all breast carcinomas exhibit *EGFR* amplification. Interestingly, one of these 11 cases was a spindle cell metaplastic carcinoma with focal squamous differentiation. Taken together, these findings suggest that EGFR overexpression and/or gene amplification are likely to play a role in carcinomas with squamous elements and spindle cell carcinomas, but perhaps not in the other subtypes of MBC.

Because the methods used by Bhargava and coworkers [8] are identical to ours, a direct comparison is feasible. Taken together, our results and those of Bhargava and coworkers indicate that *EGFR* amplification is statistically more prevalent in MBCs than in other types of breast carcinoma (10/174 nonmetaplastic breast carcinomas versus 8/26 metaplastic breast carcinomas showed *EGFR* amplification; *P *< 0.001 by Fisher's exact test (two sided)).

Although EGFR amplification accounted for 37% of EGFR overexpression in the present series, the majority of cases showed no amplification. EGFR activating mutations have been described in lung cancer and in brain tumours, but these mutations have proven extremely rare in other types of cancer, including breast carcinomas [8,37]. However, Weber and coworkers [38] recently described EGFR missense mutations in sporadic and familial (*BRCA1*/*BRCA2 *related) breast cancer and demonstrated that these mutations are significantly more frequent in the latter. Therefore, further analysis of *EGFR* gene sequence in MBCs may explain the overexpression of EGFR in those cases lacking *EGFR* amplification.

An alternative mechanism for EGFR expression in MBC may be maintenance of a myoepithelial/basal phenotype. In fact, expression of EGFR is part of the definition of 'basal-like' tumours proposed by Nielsen and coworkers [39]. EGFR is consistently expressed in myoepithelial cells of the breast [40]. We [26,41,42] and others [30,31,43-45] have demonstrated that the vast majority of MBCs consistently express basal/myoepithelial markers. Furthermore, indirect evidence from a study using murine cell lines suggests that transformed myoepithelial cells may give rise to tumours with sarcomatous and carcinosarcomatous patterns, similar to those observed in spindle cell carcinomas and carcinomas with heterologous elements [46]. Therefore, one could speculate that overexpression of EGFR, without gene amplification, could simply reflect maintenance of the basal-like/myoepithelial phenotype of these lesions. Conversely, one cannot rule out that *EGFR* gene amplification is one of the genetic mechanisms whereby basal/myoepithelial differentiation pathways are activated in transformed luminal epithelial cells.

In the present series, there was no association between EGFR overexpression and DFS or OS, whereas there was a trend toward shorter DFS and OS in patients with tumours exhibiting *EGFR* amplification. Based on our results, further studies analyzing the prognostic impact of EGFR amplification in a large cohort of MBCs are warranted.

The present study also confirms the results of previous analyses demonstrating lack of HER2 overexpression in MBCs [12,47], suggesting that humanized monoclonal antibodies against HER2 play little or no role in the treatment of these lesions.

## Conclusion

Although initial studies suggested that only tumours harbouring *EGFR* mutations would be sensitive to EGFR tyrosine kinase inhibitors, there are compelling data to suggest that tumours harbouring EGFR amplification may also respond well to these new agents [22]. Given that MBCs lack oestrogen receptor, show EGFR overexpression and harbour *EGFR* amplification in up to 28% of cases, and that some MBCs have proven refractory to standard types of treatment, the use of EGFR tyrosine kinase inhibitors may represent an alternative therapeutic regimen for patients with MBC. Further studies addressing the efficacy of EGFR inhibitors in this group of breast carcinomas are warranted.

## Abbreviations

CISH = chromogenic *in situ *hybridization; DFS = disease-free survival; EGFR = epidermal growth factor receptor; HER = human epidermal growth factor receptor; MBC = metaplastic breast carcinoma; OS = overall survival.

## Competing interests

The authors declare that they have no competing interests.

## Authors' contributions

JSR-F drafted the manuscript and participated in the histological review, immunohistochemical analysis and study design. FM participated in the histological review, immunohistochemical and CISH analyses. SC carried out the CISH analysis. PTS helped to draft the manuscript and participated in the study design. DS and KS performed the immunohistochemical reactions. ML performed part of the CISH experiments. EMP and JN participated in the histological review. SRL and FCS conceived the study, participated in its design and coordination, and helped to draft the manuscript. FCS also participated in the histological review. All authors read and approved the final manuscript.

## Figures and Tables

**Table 1:** Summary of histological, immunohistochemical and chromogenic *in situ *hybridization findings

Case	Histological type	HER2 (Herceptest^®^)	HER2 (CB11)	EGFR (IHC)	*EGFR* (CISH)
1	Carcinoma with squamous metaplasia	-	-	3+	No amp
2	Carcinoma with squamous metaplasia	-	-	3+	No amp
3	Carcinoma with squamous metaplasia	-	-	3+	No amp
4	Carcinoma with squamous metaplasia	-	-	3+	No amp
5	Carcinoma with squamous metaplasia	-	-	3+	Amp
6	Carcinoma with squamous metaplasia	-	-	3+	Amp
7	Carcinoma with squamous metaplasia	1+	-	3+	No amp
8	Carcinoma with squamous metaplasia	-	-	3+	Amp
9	Carcinoma with squamous metaplasia	-	-	1+	No amp
10	Spindle cell carcinoma	-	-	3+	No amp
11	Spindle cell carcinoma	-	-	3+	No amp
12	Spindle cell carcinoma	-	-	1+	No amp
13	Spindle cell carcinoma	-	-	3+	Amp
14	Spindle cell carcinoma	-	-	-	No amp
15	Spindle cell carcinoma	-	-	3+	Amp
16	Spindle cell carcinoma	-	-	-	No amp
17	Spindle cell carcinoma	-	-	3+	Amp
18	Spindle cell carcinoma	-	-	3+	No amp
19	Spindle cell carcinoma	2+^a^	1+^a^	3+	Amp
20	Carcinoma with heterologous elements	-	-	3+	No amp
21	Carcinoma with heterologous elements	-	-	3+	No amp
22	Matrix producing carcinoma	-	-	1+	No amp
23	Matrix producing carcinoma	-	-	-	No amp
24	Matrix producing carcinoma	-	-	3+	No amp
25	Matrix producing carcinoma	-	-	3+	No amp

^a^No *HER2* gene amplification was detected by CISH. Amp, amplification; CISH, chromogenic *in situ *hybridization; EGFR, epidermal growth factor receptor; HER, epidermal growth factor receptor; IHC, immunohistochemistry.

**Table 2:** Summary of the associations between clinicopathological parameters and EGFR overexpression and amplification

Parameter	EGFR IHC		*P*	*EGFR* CISH		*P*
				
	Negative	Overexpression		No	Amplified	
Age (years)	54.2 (13.3)	48.8 (13.3)	>0.05	50.4 (13.7)	48.8 (12.7)	>0.05
Size (cm)	4.0 (3.2)	4.4 (3.1)	>0.05	3.8 (2.9)	5.9 (3.9)	>0.05
Lympho-vascular invasion						
No	2	9	>0.05	9	2	>0.05
Yes	2	9		7	4	
Lymph node metastasis at diagnosis						
No	3	11	>0.05	10	4	>0.05
Yes	1	5		4	2	

CISH, chromogenic *in situ *hybridization; EGFR, epidermal growth factor receptor. IHC, immunohistochemistry.
